# Education paths in neuro-oncology: combining technical skills with multidisciplinary care. A survey from the AINO (Italian Association for Neuro-Oncology) Youngster Committee

**DOI:** 10.1007/s11060-025-05003-2

**Published:** 2025-03-18

**Authors:** Rina Di Bonaventura, Denis Aiudi, Silvia Chiesa, Alessia Pellerino, Francesco Bruno, Valeria Internò, Ciro Mazzarella, Edoardo Pronello, Roberto Colasanti, Teresa Somma, Tamara Ius, Giuseppe Maria Della Pepa, Valeria Barresi, Quintino Giorgio D’Alessandris, Roberta Rudà, Antonio Silvani

**Affiliations:** 1 Department of Neurosurgery, Fondazione Policlinico Universitario A. Gemelli IRCCS, Largo Agostino Gemelli 8, Rome, 00168 Italy; 2 Neurosurgical Department, Università Politecnica Delle Marche, Marche General University Hospital, Ancona, Italy; 3 Gemelli Advanced Radiotherapy, Fondazione Policlinico Universitario A. Gemelli IRCCS, Rome, Italy; 4 Division of Neuro-Oncology, Department of Neuroscience ʺRita Levi Montalciniʺ, City of Health and Science Hospital and University of Turin, Turin, Italy; 5 Oncology Unit, San Paolo Hospital, Bari, Italy; 6 Department of Neurosurgery, Maurizio Bufalini Hospital, AUSL della Romagna, Cesena, Italy; 7 Division of Neurosurgery, Department of Neurological Sciences, Università Degli Studi Di Napoli Federico II, Naples, Italy; 8 Academic Neurosurgery, Department of Neurosciences, University of Padova, Padova, Italy; 9 Department of Diagnostics and Public Health, University of Verona, Verona, Italy; 10 Pathology-Neuropathology Unit, Fondazione IRCCS Istituto Neurologico Carlo Besta, Milan, Italy; 11 Department of Neuroscience, Neurosurgery section, Università Cattolica del Sacro Cuore, Rome, Italy; 12 Neuro-Oncology Unit, Fondazione IRCCS Istituto Neurologico Carlo Besta, Milan, Italy

**Keywords:** Neuro-oncology, Education, Training, Mentorship, Multidisciplinary care

## Abstract

**Purpose:**

Neuro-oncology is a multidisciplinary subspecialty that has evolved and expanded tremendously over the last 20 years. In Europe, notwithstanding a number of commendable initiatives, neither a specific neuro-oncology training curriculum nor a consensus on the ideal training tools have been set. In this context, the Youngster Committee of the Italian Association for Neuro-Oncology (AINO) has run a nationwide survey to take a snapshot of the current situation of neuro-oncology education in Italy.

**Methods:**

Between July and November 2023, we distributed through AINO a 34-question survey addressed to all Italian care providers dealing with neuro-oncology, irrespective of specialty and level of experience, as per AINO mission. The questionnaire was disseminated using an open link. We analyzed and stratified answers according to epidemiological characteristics of the respondents, i.e. age, gender, role, years of experience, type and case load of their work Institutions, geographical region.

**Results:**

We collected 254 valid questionnaires. The majority of respondents were under 40 years old (62.6%); neurosurgeons formed the largest specialty group (48%). Residency was a key step for neuro-oncology education according to 33% of participants; notably, younger respondents gave a significantly more positive assessment of residency programs compared to older ones (72% vs. 56%, *p* = 0.0193). PhD programs in Italy are focused only on research, according to 30% of respondents. Regarding the tools for continuing medical education in neuro-oncology, a striking contrast between the ideal ones, which should be the frequent participation in dedicated courses (59% responses), and the actual one, which is scientific literature (55%), was recorded. Mentorship programs are rare and inconsistent and should be strengthened. More than 90% of participants declared multidisciplinary collaboration as fundamental. Multispecialty societies like AINO have a key role in strengthening education in neuro-oncology through the organization of structured post-graduate programs.

**Conclusion:**

The results of this survey, by describing the status of the neuro-oncology training paths in Italy, can lay the foundation for initiatives aimed at harmonizing neuro-oncology education in Italy and Europe. The creation of a shared neuro-oncology curriculum and of a network of mentors is suggested.

**Supplementary Information:**

The online version contains supplementary material available at 10.1007/s11060-025-05003-2.

## Introduction

Neuro-oncology is a relatively recent but rapidly evolving clinical and research field dealing with nervous system tumors [1]. It is inherently multidisciplinary, integrating contributions from both organ-specific and non-organ-specific specialties. Unfortunately, although specialized training in neuro-oncology is crucial for enhancing proficiency and elevating the standard of care [1], exposure to neuro-oncology remains limited in most medical schools and residency programs [2]. The optimal training in neuro-oncology for the involved specialties has long been debated. In the United States, a yearly fellowship training program, accredited by the United Council for Neurologic Subspecialties (UCNS), is available, and it is followed by UCNS certification [3, 4]. Fellowship and board certification are open to physicians coming from different specialties, including neurology, child neurology, neurological surgery, internal medicine, medical oncology, pediatrics and pediatric hematology-oncology, and radiation oncology [2]. In Europe, such a structured fellowship program is lacking; however, numerous courses and educational paths have been established by multidisciplinary neuro-oncology societies and by the neuro-oncology sections of single-specialty societies [5]. In 2022, the European Association for Neuro-Oncology (EANO) launched its “School of Neuro-Oncology”, “a comprehensive high level, postgraduate, in-depth two-year virtual programme” [6]. A three-year mentorship program by the European Organisation for Research and Treatment of Cancer is also in place.

In Italy, neuro-oncology care is provided by different specialists, with some local variations. The core members of multidisciplinary neuro-oncology boards are medical oncologists, neurologists, neurosurgeons, pathologists, radiation oncologists and radiologists. The Italian Association for Neuro-Oncology (AINO) gathers all healthcare professionals involved in care and research on neurological tumors; AINO is the Italian member of the World Federation of Neuro-Oncology Societies, and it is an EANO partner. As previously stated, no structured educational programs in neuro-oncology have been set up, though many commendable initiatives are in place. In this scenario, the Youngster Committee of AINO conducted a survey to explore the paths of education in neuro-oncology in Italy, aiming to take a snapshot of the current landscape, recognize achieved milestones, and identify future goals for development.

## Methods

### Conception and diffusion of the survey

The survey was drafted during a dedicated meeting of the AINO Youngster committee and was then approved by the AINO Executive Board. The main topics that were considered for inclusion in the survey were as follows: education background of care providers in neuro-oncology, continuous medical education (CME) in neuro-oncology, multidisciplinary care and mentorship in neuro-oncology, and the role of multidisciplinary societies like AINO in promoting neuro-oncology education. The final questionnaire was distributed online in anonymous form and in the Italian language between July and November 2023, using an open link. All care providers involved in neuro-oncology, irrespective of their age, role in the team and level of experience, were considered eligible for the survey, according to AINO spirit and mission. The dissemination of the survey was fostered by the collaboration of several Italian scientific societies, including AINO, the Italian Society for Neurosurgery (SINch) and its Youngster Committee, the Italian Association for Radiation Oncology (AIRO), and the Italian Neuropathology Group (GINP) of the Italian Society of Pathologic Anatomy and Cytopathology (SIAPEC). About 1300 links were directly sent to healthcare providers involved in neuro-oncology; neurosurgeons formed the largest group. No formal control for authenticity or duplicate data was in place.

Data from responses were collected and managed using REDCap (Research Electronic Data Capture) tools hosted at Fondazione Policlinico Universitario A. Gemelli IRCCS [7, 8]. REDCap is a secure, web-based software platform designed to support data capture for research studies. Consent was requested from all survey participants.

### Structure of the survey

The survey questionnaire consisted of 34 questions divided into four parts, namely: *Participants demographics and job details*, *Neuro-oncology education*, *Neuro-oncology personal vision*, *Perspectives*. Overall, there were 29 multiple-choice questions, of which 25 allowed for a single response and four for multiple responses. Four questions required ranking a list of options, and the final item of the survey was an open-ended question inviting free comments. The full questionnaire is available as Supplementary Material 1, Supplementary Methods.

### Data analysis

Categorical variables were compared using the Chi-square statistics, applying the Fisher exact test when appropriate. The RedCap platform and Microsoft Excel were used for data and graphs elaboration, while MedCalc ver 20.015 (MedCalc Software Ltd., Ostend, Belgium) was used for statistical analysis. A *p* < 0.05 was considered significant.

## Results

A total of 265 records were collected on the RedCap platform as responses to the survey. Of these, 11 responses were excluded from analysis due to lack of consent (*n* = 3) or incomplete data in Part 1 of the survey (*n* = 8). Thus, the final study group consisted of 254 participants who provided consent and completed at least Part 1 (Table 1). Responses are available as Supplementary Material 2, Supplementary Tables Q0-Q34 (processed data) and Supplementary Material 3, Supplementary Table S1 (raw data).

**Table 1:** General and education characteristics of interviewees

Option	*n* (%)
M: F	127: 123 (50.0%: 48.4%)
Age	
< 40	159 (62.6%)
40–59	83 (32.7%)
≥ 60	12 (4.7%)
Years of Neuro-oncology Experience	
≤ 10	181 (71.3%)
11–20	48 (18.9%)
> 20	25 (9.8%)
Specialty	
Pathologist	33 (13.0%)
Neurosurgeon	122 (48.0%)
Neurologist	31 (12.2%)
Medical Oncologist	11 (4.3%)
Radiologist	9 (3.5%)
Radiation Oncologist	33 (13.0%)
Other	15 (5.9%)
First steps in neuro-oncology	
Before job placement	180 (80.4%)
After job placement	44 (19.6%)
Most important step of education	
Residency Program	46 (32.6%)
Fellowships in Italy or abroad	40 (28.4%)
Ph.D. Program	39 (27.7%)
Conferences and courses	16 (11.3%)
Participation to clinical Trial during training	87 (38.8%)
Active translational research during training	125 (55.8%)

### Part 1 - Characteristics of the participants

Participants’ general features are summarized in Table 1. Genders were well balanced. As for age, young physicians under 40 years comprised 62.6% of the cohort, with 71.3% having up to 10 years of experience in neuro-oncology care. Regarding specialty, half of the participants were neurosurgeons (48%), while the remainder included neurologists, radiation oncologists, pathologists and others. The survey garnered interest across Italy, with more than 40% of participants from the northern part of the country, about 30% from the central part, and about 25% from the southern or insular regions.

Table 2 provides insights into the workplace and career stages of the respondents. Most of them work in large hospitals (61.8% with a caseload of more than 100 brain tumors per year) and university/research hospitals (57.5%), with a remarkable contribution from non-teaching hospitals (31.9%). A multidisciplinary Brain Tumor board is active in 87% of institutions, with a significantly higher prevalence in university/research hospitals compared to non-teaching hospitals (91.8% vs. 76.5%, respectively; *p* = 0.002, Fisher exact test). Over 75% of participants reported that their institutions promote neuro-oncology educational meetings; the percentage is higher in university/research hospitals (82.9% vs. 61.7% in non-teaching hospitals; *p* < 0.001; Fisher exact test). The majority of respondents work as team members (52.6%), but with a notable representation of group leaders (department directors or unit heads; 13.8%). Additionally, 67% of participants are involved in neuro-oncology research, with half dedicating 10 to 30% of their work time to research activities.

**Table 2:** Interviewees’ work characteristics

Option	*n* (%)
Institution	
University/Research Hospital	146 (57.5%)
Non-teaching Hospital	81 (31.9%)
Other	27 (10.6%)
Institution caseload	
≤ 100 cases/year	97 (38.2%)
> 100 cases/year	157 (61.8%)
Presence of a multidisciplinary Tumor Board	
Yes	221 (87.0%)
No	33 (13.0%)
Presence of educational neuro-oncology meetings	
Yes	192 (75.6%)
No	62 (24.4%)
Role in working Group	
Resident/PhD Student/Fellow	71 (28.1%)
Physician	133 (52.6%)
Group Leader	35 (13.8%)
Private pratictioner	14 (5.5%)
Involved in research	170 (66.9%)

### Part 2 – Neuro-oncology training and education

**Fig. 1:**
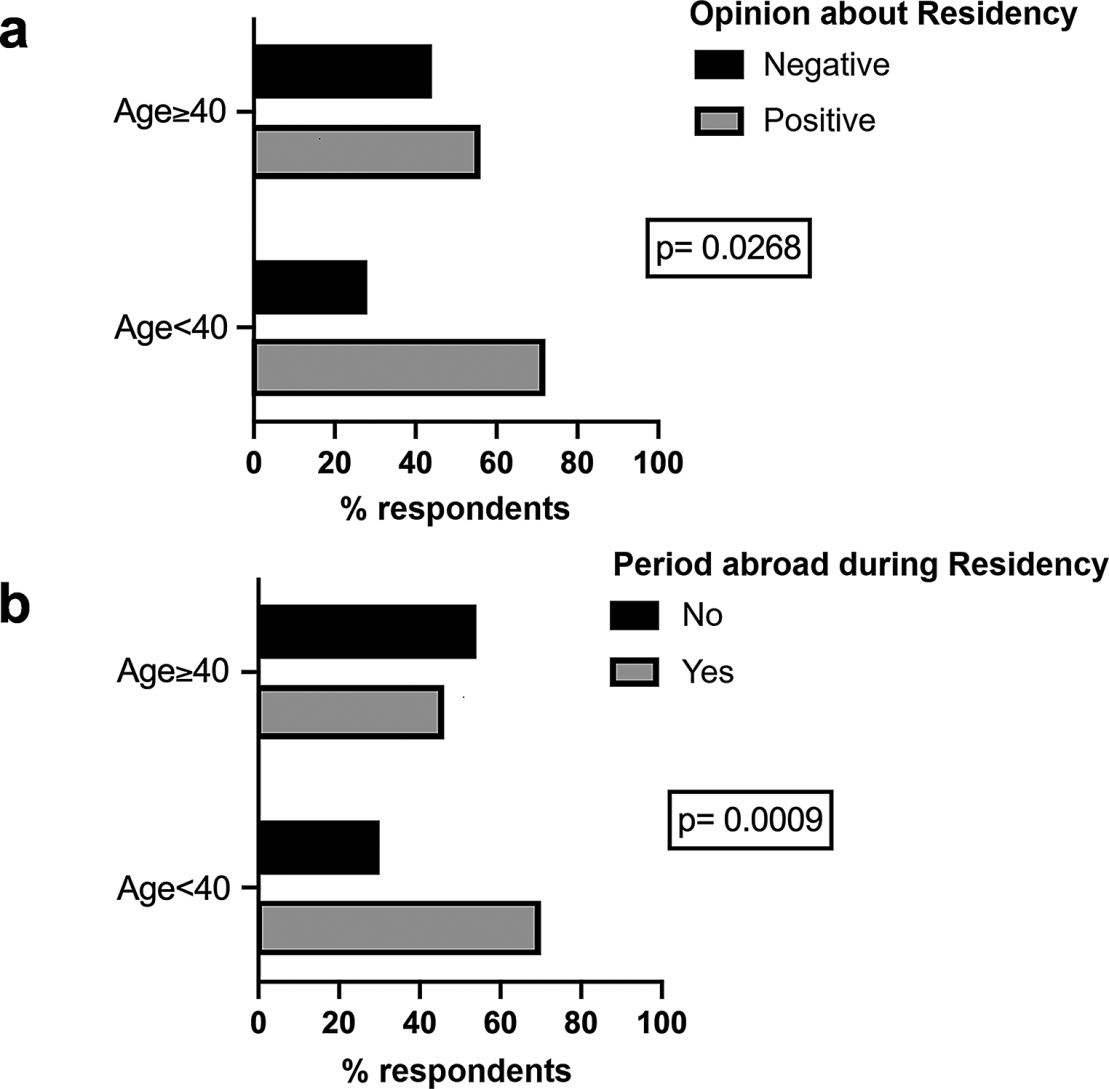
Quality of residency programs. **a**, overall evaluation depending on age. **b**, presence of a period abroad during residency depending on age

The majority of participants stated that their involvement in neuro-oncology care stemmed from a spontaneous vocation (61.6%; Supplementary Material 2, Supplementary Table Q15). Neuro-oncology careers started before job placement for 80.4% of respondents, and 32.6% recognized residency as the most crucial step in neuro-oncology education (Table 1). In agreement with this finding, 66.2% of participants expressed a positive opinion about their residency school, with those under 40 years old reporting significantly better evaluations than their older counterparts (72.1% positive evaluation rate for participants < 40 vs. 56.0% for those ≥ 40; *p* = 0.0193, Fisher Exact Test; Fig. 1a and Supplementary Material 2, Supplementary Table Q18A). Most respondents identified the specific technical preparation as the main strength of Italian residency schools (73.7%; Supplementary Material 2, Supplementary Table Q19). However, only 51.8% felt that their residency program adequately prepared them for multidisciplinary interaction, and about one-third expressed concerns regarding inadequate preparation to manage stress related to patient care (Supplementary Material 2, Supplementary Table Q20).

As for PhD programs, 35.1% of participants believed that PhDs adequately prepared them for both research and clinical neuro-oncology; conversely, 30% of respondents stated that the only focus of PhD programs was research activity (Supplementary Material 2, Supplementary Table Q21).

A period abroad during residency training was reported by 61.2% of the participants overall. This figure was significantly higher in respondents under 40 years old compared to older respondents (70% vs. 46%, respectively; *p* = 0.0006, Fisher exact test; Fig. 1b and Supplementary Material 2, Supplementary Table Q23A). Most participants (55.8%) gained experience in basic or translational research during their training (Table 1 and Supplementary Material 2, Supplementary Table Q24), while only 38.8% reported participating in a clinical trial (Table 1 and Supplementary Material 2, Supplementary Table Q25).

Regarding Continuing Medical Education (CME) in Neuro-Oncology, the main actual resource was scientific literature (54.9%) (Supplementary Material 2, Supplementary Tables Q22 and Q22A).

### Part 3 – Your vision of neuro-oncology

Dealing with the ideal modalities for CME in neuro-oncology, the majority of participants (59.3%), across all age groups, institutions, and specialties, recognized the importance of attending dedicated courses and meetings, possibly more than once a year, while relying solely on scientific literature was judged sufficient by less than 10% of respondents (Supplementary Material 2, Supplementary Tables Q26–Q26C).

As expected, multidisciplinary collaboration was reported as fundamental by more than 90% of the respondents, with no significant differences observed across age groups (< 40 vs. ≥ 40), institution types (non-teaching vs. teaching hospitals) or specialties (*p* = 0.31, Fisher exact test; *p* = 0.17, Fisher exact test; and *p* = 0.16, Chi-square test, respectively; Supplementary Material 2, Supplementary Tables Q27–Q27C).

**Fig. 2:**
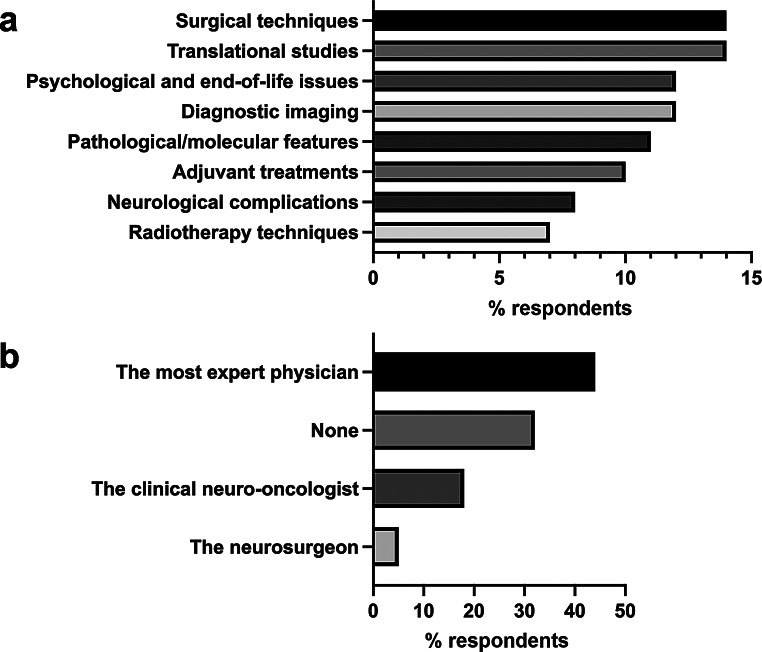
Multidisciplinary care in neuro-oncology according to survey participants. **a**, core neuro-oncology abilities. **b**, leader of the multidisciplinary neuro-oncology group

With the aim of getting to the core of neuro-oncology transversal knowledge, the survey asked respondents to rank essential skills to be gained by all physicians involved in neuro-oncology care during their training. Surgical techniques and the ability to conduct translational studies were rated highest (14.2% each; Fig. 2a and Supplementary Material 2, Supplementary Table Q28A). Pathologists, neurologists, and radiation oncologists mostly contributed to the high scoring of surgical techniques, while translational studies received appreciation across all specialties (Supplementary Material 2, Supplementary Table Q28D).

Furthermore, we inquired whether the multidisciplinary board should have a designated leader. As shown in Fig. 2b and Supplementary Material 2, Supplementary Table Q29, 32% of the respondents believed that there should not be a leader, while the majority (44.1%) answered that the most experienced physician, regardless of specialty, should lead. Interestingly, younger respondents (< 30 years old) were more likely to advocate for an experienced leader (52%), in contrast to respondents over 60 years old, who mostly did not consider a leader necessary (40%, Online resource 2, Supplementary Table Q29A). Additionally, responses varied among different specialists, with pathologists, neurosurgeons, and medical oncologists favoring an experienced leader, while radiologists and radiation oncologists preferring no leader (Online resource 2, Supplementary Table Q29C). These differences were not statistically significant.

### Part 4 – Perspectives in neuro-oncology

**Fig. 3:**
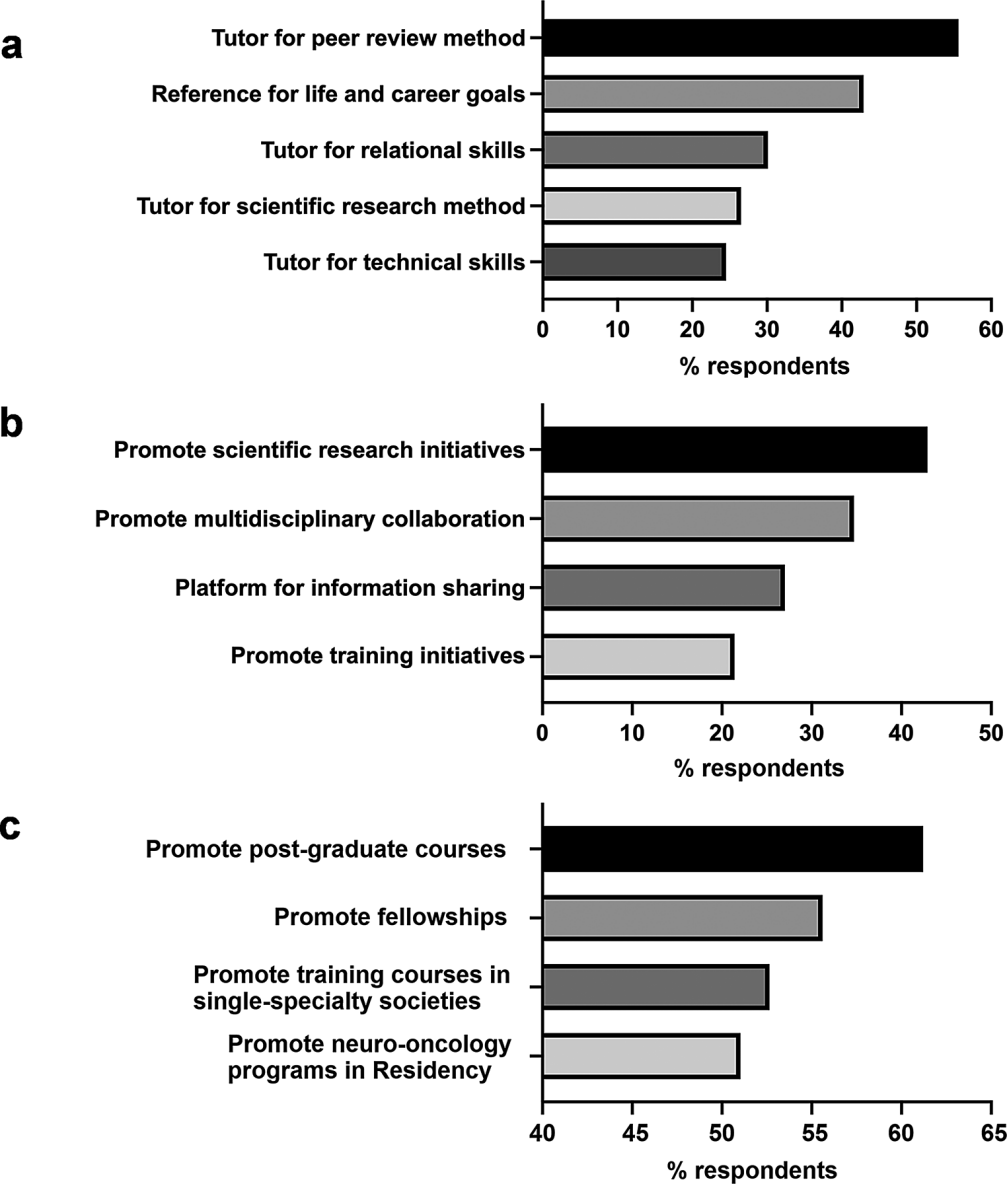
Perspectives in neuro-oncology education according to survey participants. **a**, role of the mentor. **b**, role of AINO Youngster Committee; **c**, role of AINO

Only 40% of respondents were aware of mentorship programs (Online resource 2, Supplementary Table Q30). This percentage remained consistent across all age groups, except for a lower figure (18.2%) in the youngest cohort (Online resource 2, Supplementary Table Q30A). Instead, respondents from teaching/research hospitals reported a significantly greater awareness of mentorship programs than those from non-teaching hospitals (47.9% vs. 23.3%; *p* = 0.0019, Fisher exact test; Online resource 2, Supplementary Table Q30B). When asked to rank the expectations from the mentor, the majority of respondents pointed to the acquisition of a rigorous peer review method (55.6%), and a reference model for establishing career goals and achieving work-life balance (42.9%) (Fig. 3a and Online resource 2, Supplementary Table Q31A). These two preferences were generally top-rated among groups regardless of age, work institution and specialty, with the following exceptions: participants older than 60, who highlighted the mentor’s role in developing specific technical skills (50%); neurologists, who emphasized the importance in acquiring relational skills (33.3%); and radiologists, who underlined the mentor’s role in developing a method for scientific research (42.9%) (Online resource 2, Supplementary Tables Q31C-E).

Questions 32 and 33 inquired into the role of AINO and the AINO Youngster Committee in endorsing neuro-oncology education. Regarding the AINO Youngster Committee, the majority of participants across all age groups highlighted its role in promoting scientific research initiatives (42.9%; Fig. 3b and Online resource 2, Supplementary Table Q32A). However, older participants and participants working in non-teaching hospitals also pointed to the role of the AINO Youngster Committee as a platform for discussing complex cases, sharing job opportunities, and promoting the culture of multidisciplinary collaboration (Online resource 2, Supplementary Tables Q32B and Q32C). As for the AINO society, the most reported request was the promotion of post-graduate neuro-oncology courses (61.2%; Fig. 3c and Online resource 2, Supplementary Table Q33), with younger participants advocating for dedicated neuro-oncology programs in specialty schools (68.2% of participants under 30 years old).

Free proposals suggested in the last question of the survey included: dedicated programs in neuro-oncology during residency, fellowship programs, working groups, and support from philanthropic organizations for neuro-oncology education and research (Online resource 2, Supplementary Table Q34).

## Discussion

The survey aimed at providing a snapshot of the current situation of neuro-oncology education in Italy, while also gathering insights on the perception of neuro-oncology as a multidisciplinary field and soliciting constructive suggestions for improving current practice.

The survey obtained a substantial number of responses from physicians across various institutions, thus demonstrating the vitality of the Italian neuro-oncology community. Moreover, the survey revealed a wide distribution of multidisciplinary neuro-oncology meetings (almost 90%) and educational meetings (over 75%) across Italian healthcare institutions. Tumor boards are perceived as crucial platforms for sharing knowledge on cutting-edge technologies and ongoing clinical trials, as well as for discussing challenging clinical cases in order to customize treatments [9–11].

Post-graduate medical education in Italy consists of residency schools and PhD programs, both university-based. Residency school in Medical Oncology lasts 5 years and encompasses clinical duties, scientific activities, grand rounds and seminars. The acquisition of skills and knowledge is assessed through yearly exams and the discussion of a final thesis. After certification in Medical Oncology, one is entitled to work in public or private settings. A similar organization is in place for the other residency schools. PhD programs are intended to provide “the skills needed to perform […] highly qualified research activities (decree of the Italian Ministry of University and Research, no 226/2021) and are organized in a more flexible way.

The majority of participants (66.2%) expressed a positive judgment on neuro-oncology training during residency, with a significantly higher rate of positive evaluations among younger participants compared to older participants. Accordingly, younger participants reported a period abroad during residency significantly more frequently than older participants did. PhD programs, instead, still face limitations, since their understandable primary focus on research comes at the expense of a missing enhancement of clinical neuro-oncology skills.

A key weakness identified was neuro-oncology CME. While most responders agreed on the opportunity to participate in more than one meeting/course per year, the most widespread current modality for CME remains scientific literature. This finding reflects a gap between ideal and actual educational practices, likely due to limited resources for attendance and difficulties in securing time off for education.

We must acknowledge that respondents found it difficult to correctly answer the question regarding the main skills that should be learned during neuro-oncology training, leading to results that should be interpreted cautiously. To note, suggesting a “core curriculum” for neuro-oncology was out of the scope of the present survey. A dedicated survey and a consensus paper on core competencies in surgical neuro-oncology have been recently published by the European Association of Neurosurgical Societies (EANS) neuro-oncology section [9, 10], and single-specialty societies (e.g., European Society for Radiotherapy and Oncology, ESTRO) have proposed neuro-oncology core curricula [12]. In any case, a core curriculum must be supported by shared practice and debate [13, 14], which are invaluable in acquiring the right blend of clinical intuition, pattern recognition, and the ability to adapt and react to disruptions in expected patterns that distinguish a real expert [10].

The survey results showed that all neuro-oncology care providers are aware of the essential value of multidisciplinary care; this was also reflected by the speculation about the “leader” of the neuro-oncology multidisciplinary group, who should not exist according to a substantial number (32%) of participants.

On the contrary, the concept of mentorship in neuro-oncology is somewhat novel and of uncertain meaning within the Italian community, where mentorship programs are still rare. According to current literature, mentoring has a crucial role in the development of the next generation neuro-oncologists. Mentoring should primarily aim to promote career progression, research, clinical skills, and clinical confidence. Moreover, a mentor should give advice on the balance between clinical practice and research, and work with personal life [15]. Mentoring in neuro-oncology should also focus on developing abilities for interdisciplinary collaboration, communication, and ethical judgment. The European Resident and Research Fellow Section of the European Academy of Neurology (EAN) conducted a survey in 2017, which acted upon an initial proposal prompting the EAN to create a mentorship program. Such initiatives should be emulated at the country level to create a network of education in neuro-oncology.

Finally, the survey pointed out the potential role of AINO in strengthening education in neuro-oncology. Structured post-graduate programs or formal fellowships could be considered, encompassing also those residents willing to be involved in neuro-oncology care.

The main limitation of this survey concerns the imbalance in distribution among participants regarding age, with 49.2% between 30 and 39 years old, and specialty, with 48% neurosurgeons. Such an imbalance could have substantially altered the current scenario and the needs of neuro-oncology education that emerged from this survey. The diffusion of the survey among different specialists of different ages and levels of expertise can also be regarded as a limitation. Another limitation was the difficulty in ranking responses in the multiple-choice questions. Finally, the survey was distributed only in Italy, and the picture taken could not reflect the situation in other countries. On the other hand, the large sample size within the Italian neuro-oncological community, and the adequate representation of physicians across different institution types, are strong points of the present study.

## Conclusions

Decades have passed since the first Conference on Brain Tumor Research and Therapy, which attracted only 35 attendees; [16] today, the neuro-oncological community is both numerous and rapidly growing worldwide. Given the specialized nature of neuro-oncology, dedicated training is needed. This survey scrutinized the current situation of education in neuro-oncology in Italy, highlighting its strengths and weaknesses to gather proposals for improvement. A feasible agenda could involve the establishment of a certified neuro-oncology curriculum and the development of a robust network of mentors [17].

## Electronic supplementary material

Below is the link to the electronic supplementary material.

**Supplementary Material 1:** Supplementary Methods

**Supplementary Material 2:** Supplementary Tables

**Supplementary Material 3:** Supplementary Table S1

## Data Availability

The dataset generated during the current study is available as Online Resource 3, Supplementary Table S1.

## References

[1] Levin VA (1999) Neuro-oncology: an overview. Arch Neurol 56(4):401-4. 10.1001/archneur.56.4.401. PMID: 10199326 10.1001/archneur.56.4.401 10199326

[2] Gonzalez Castro LN, Forst DA, Plotkin SR, Lee EQ (2022) A practical guide to neuro-oncology fellowship. J Neurooncol 156(1):73–79. 10.1007/s11060-021-03897-2. Epub 2021 Dec 2. PMID: 34855097 34855097 10.1007/s11060-021-03897-2

[3] Barbaro M, Fine HA, Magge RS (2021) Foundations of Neuro-Oncology: A multidisciplinary approach. World Neurosurg 151:392–401. 10.1016/j.wneu.2021.02.059Epub 2021 Feb 20. PMID: 33618043 33618043 10.1016/j.wneu.2021.02.059

[4] Jensen R, O’Rourke D, Warnick R, Sawaya R (2006) Resident training in neurosurgical oncology: results of the survey of North American training programs by the AANS/CNS Section on Tumors. J Neurooncol 77(3):241-6. 10.1007/s11060-005-9043-7. PMID: 16544056 10.1007/s11060-005-9043-7 16544056

[5] Gonzalez Castro LN, Forst DA, Plotkin SR, Lee EQ (2022) A practical guide to neuro-oncology fellowship. J Neurooncol 156(1):73–79. 10.1007/s11060-021-03897-2. Epub 2021 Dec 2. PMID: 34855097 10.1007/s11060-021-03897-2 34855097

[6] https://www.eano.eu/school-of-neuro-oncology/general-information/, accessed August 14, 2024

[7] Harris PA, Taylor R, Minor BL, Elliott V, Fernandez M, O’Neal L, McLeod L, Delacqua G, Delacqua F, Kirby J, Duda SN, REDCap Consortium (2019) The REDCap consortium: Building an international community of software platform partners. J Biomed Inf 95:103208. 10.1016/j.jbi.2019.103208Epub 2019 May 9. PMID: 31078660; PMCID: PMC7254481 10.1016/j.jbi.2019.103208 PMC7254481 31078660

[8] Harris PA, Taylor R, Thielke R, Payne J, Gonzalez N, Conde JG (2009) Research electronic data capture (REDCap)--a metadata-driven methodology and workflow process for providing translational research informatics support. J Biomed Inf 42(2):377–381. 10.1016/j.jbi.2008.08.010 Epub 2008 Sep 30. PMID: 18929686; PMCID: PMC2700030 10.1016/j.jbi.2008.08.010 PMC2700030 18929686

[9] Gousias K, Hoyer A, Mazurczyk LA, Bartek J Jr, Bruneau M, Celtikci E, Foroglou N, Freyschlag C, Grossman R, Jungk C, Metellus P, Netuka D, Rola R, Schucht P, Senft C, Signorelli F, Vincent AJPE, Simon M (2024) EANS surgical Neuro-oncology expertise survey working group. expertise in surgical neuro-oncology. Results of a survey by the EANS neuro-oncology section. Brain Spine 7(4):102822. 10.1016/j.bas.2024.102822PMID: 38831935; PMCID: PMC11145419 10.1016/j.bas.2024.102822 PMC11145419 38831935

[10] Kamp MA, Malzkorn B, von Sass C, DiMeco F, Hadjipanayis CG, Senft C, Rapp M, Gepfner-Tuma I, Fountas K, Krieg SM, Neukirchen M, Florian IȘ, Schnell O, Mijderwijk HJ, Perin A, Baumgarten P, van Lieshout JH, Thon N, Renovanz M, Kahlert U, Spoor JKH, Hänggi D, McLean AL, Mäurer M, Sarrubbo S, Freyschlag CF, Schmidt NO, Vergani F, Jungk C, Stein M, Forster MT, Weinberg JS, Sinclair J, Belykh E, Bello L, Mandonnet E, Moiyadi A, Sabel M (2021) Proposed definition of competencies for surgical neuro-oncology training. J Neurooncol 153(1):121–131. 10.1007/s11060-021-03750-6Epub 2021 Apr 21. PMID: 33881726; PMCID: PMC8131302 33881726 10.1007/s11060-021-03750-6 PMC8131302

[11] Snyder J, Schultz L, Walbert eT (2017) The Role of Tumor Board Conferences in Neuro-Oncology: A Nationwide Provider Survey. *Journal of Neuro-Oncology* 133(1): 1–7. 10.1007/s11060-017-2416-x 10.1007/s11060-017-2416-x 28421461

[12] Garibaldi C, Essers M, Heijmen B, Bertholet J, Koutsouveli E, Maas AJJ, Moore M, Petrovic B, Koniarova I, Lisbona A, Piotrowski T, Moeckli R, López Medina A, Stylianou Markidou E, Clark CH, Jornet N (2021) Towards an updated ESTRO-EFOMP core curriculum for education and training of medical physics experts in radiotherapy - A survey of current education and training practice in Europe. Phys Med 84:65–71. 10.1016/j.ejmp.2021.03.030Epub 2021 Apr 13. PMID: 33862451 33862451 10.1016/j.ejmp.2021.03.030

[13] Benstead K, Brandl A, Brouwers T, Civera J, Collen S, Csaba DL, De Munter J et al (2023) An Inter-Specialty Cancer training programme curriculum for Europe. Eur J Surg Oncol 49(9):106989. 10.1016/j.ejso.2023.106989 37556988 10.1016/j.ejso.2023.106989

[14] Kleineberg NN, Van Der Meulen M, Franke C, Klingelhoefer L, Sauerbier A, Di Liberto G, Carvalho V, Berendse HW, Deuschl eG (2020) Differences in neurology residency training programmes across Europe – a survey among the residents and research fellow section of the European academy of neurology National representatives. Eur J Neurol 27(8):1356–1363. 10.1111/ene.14242 32248603 10.1111/ene.14242 PMC7496990

[15] Akhigbe T, Zolnourian A, Bulters D (2017) Mentoring models in neurosurgical training: review of literature. J Clin Neurosci 45:40–43. 10.1016/j.jocn.2017.07.025Epub 2017 Aug 12. PMID: 28811078 28811078 10.1016/j.jocn.2017.07.025

[16] Berg P (2008) Meetings that changed the world: Asilomar 1975: DNA modification secured. Nature 18;455(7211):290-1. 10.1038/455290a. PMID: 18800118 10.1038/455290a 18800118

[17] Weller M (2018) Next generation neuro-oncology. Eur J Cancer 96:1–5. 10.1016/j.ejca.2018.03.016. Epub 2018 Apr 12. PMID: 29656021 10.1016/j.ejca.2018.03.016 29656021

