# Supplementary material for: Education paths in neuro-oncology: combining technical skills with multidisciplinary care. A survey from the AINO (Italian Association for Neuro-Oncology) Youngster Committee
Source: J Neurooncol. 2025 Mar 18;173(2):469–77. doi: 10.1007/s11060-025-05003-2 (PMC12106497; doi:10.1007/s11060-025-05003-2)
Supplement: Supplementary file 1 — Supplementary Material 1: Supplementary Methods [file 11060_2025_5003_MOESM1_ESM.pdf]

Education Paths in Neuro-Oncology: combining technical skills with multidisciplinary care.
A survey from the AINO (Italian Association for Neuro-Oncology) Youngster Committee

The numbers of the options correspond to the response code in Supplementary Table S1.

Would you give your consent to participate in this survey?
0- No
Missing- No
1- Yes

PART 1 - CHARACTERISTICS OF THE PARTICIPANT
1) Age (years)
1- <30
2- 30–39
3- 40–49
4- 50–59
5- ≥ 60

2) Gender
1- Female
2- Male
9- I prefer not to specify

3) Which is your specialty?
1- Pathologist
2- Neurosurgeon
3- Neurologist
4- Neuropsychologist
5- Medical Oncologist
6- Radiologist
7- Radiation Oncologist
9- Other

4) How many years have you been involved in neuro-oncology?
1- Less than 5
2- 5–10
3- 11-20
4- More than 20

5) What type of Institution do you work for?
1- General Hospital
2- University Hospital
3- Research Hospital (IRCCS)
4- Mixed
5- Private pratictioner

6) Where do you work?

1- North
2- Centre
3- South
4- Islands
5- Abroad
- If you want, specify the region

7) Where did you carry out your postgraduate education?
1- North
2- Centre
3- South
4- Islands
5- Abroad
- If you want, specify the region

8) What is your role in the working group?
1- Resident
2- PhD student/ research fellow
3- Physician
4- Unit chief
5- Director
6- Private practitioner
7- University Professor

9) What is the neuro-oncological case load of your Institution, including new diagnoses and follow-up?
1- Less than 50 cases
2- 50–100 cases
3- 100–500 cases
4- More than 500 cases

10) Does Neuro-oncology Tumor Board routinely operate at your Institution?
1- Yes, with weekly or multi-weekly meetings
2- Yes, with fortnightly meetings
3- Yes, with monthly meetings
4- No

11) Does your Institution organize educational neuro-oncology meetings?
1- Yes, on a regular basis, at least once a month;
2- Yes, on a regular basis, less than once a month;
3- Yes, occasionally;
4- No.

12) Are you actively involved in neuro-oncology research?
1- Yes
0- No

13) If Yes, what kind of research?

1- Surgical research;
2- Clinical research;
3- Translational research.

14) Which percentage of your work time do you spend on research activities?
1- <10%
2- 10–30%
3- 30–50%
4- >50%

PART 2 – NEURO-ONCOLOGY TRAINING AND EDUCATION
15) What is the main reason of your involvement in neuro-oncology?
1- Spontaneous vocation
2- Legacy of the mentor
3- Need of the Istitution you work for

16) At what stage of your career did you start dealing with neuro-oncology?
1- Medical Student
2- Resident
3- PhD student/research fellow
4- After job placement

17) What were the most important steps in your neuro-oncology education? Please rank options from the most important (1) to the least important (4) (9:N/A).
1- Residency Program
2- Ph.D. Program
3- Fellowships in Italy or abroad
4- Conferences and courses

18) Do you believe that the Residency Program you attended adequately prepared you for your neuro-oncology activity?
1- Yes, in a complete and exhaustive way
2- Sufficiently
3- Scarcely
4- Not at all

19) What were the strength points of your Residency Program in the neuro-oncology field?
1- Technical and scientific preparation to deal with neuro-oncological diseases
2- Preparation to manage the psychological stress and care load caused by dealing with the neuro-oncological patient
3- Preparation to manage multidisciplinary neuro-oncological interaction
9- Other

20) What were the weaknesses and/or aspects to be improved of your Residency Program in the neuro-oncology field?
1- Insufficient technical and scientific preparation to deal with neuro-oncological diseases
2- Insufficient preparation to manage the psychological stress and care load caused by the neuro-oncological patient

3- Insufficient preparation to manage multidisciplinary neuro-oncological interaction
9- Other

21) If you have attended a PhD Program, do you think it has prepared you for clinical neuro-oncology activity?
1- Very much
2- Sufficiently
3- Scarcely
0- No: the PhD Program only prepared me for neuro-oncology research
9- I did not attend a PhD Program

22) What is your main tool for Continuing Neuro-Oncology Education?
1- National Meetings
2- International Meetings
3- Masters/scholarships/ courses
4- Tumor board
5- Scientific literature
6- Daily clinical practice

23) Do your Residency or Ph.D. Programs include a period abroad to deepen the knowledge of neuro-oncological topics of interest?
1- Yes, but short-lived (<3 months)
2- Yes, up to 6 months
3- Yes, from 6 to 12 months
4- Yes, more than 1-year long
0- No

24) During your training, did you have experience in basic/translational research?
1 - Yes
0 – No

24*) If not, would you have been interested in?
1- Yes
0- No

25) During your training, have you participated in clinical trials as subinvestigator or study coordinator?
1-Yes
0- No

25bis)If not, would you have been interested in?
1- Yes
0- No

PART 3 – YOUR VISION OF NEURO-ONCOLOGY
26) What are the ideal modalities and frequency of Continuous Neuro-oncology Education, in your opinion?
1- Participation in at least one neuro-oncology meeting/course per year

2- Participation in more than one neuro-oncology meeting/course per year
3- It is sufficient to keep up to date with the guidelines and literature
0- I do not think a particular continuous education schedule is necessary.

27) The multidisciplinary collaboration in neuro-oncology is, in your opinion:
1- Fundamental: care program must always come from the multidisciplinary group
2- Useful, but the referring specialist retains the path of care of his patient
3- Optional, as each specialist must do his or her work independently of the others.

28) The multidisciplinary neuro-oncology collaboration requires that each specialist gains a certain degree of knowledge of technical aspects concerning specializations other than his/hers. Please rank the following skills according to what you think are the most important to define yourself as a neuro-oncology care provider (1: most important; 8: least important; 9:N/A)
1- Diagnostic imaging of nervous system tumors
2- Surgical techniques for nervous system tumors resection
3- Pathological/molecular features of nervous system tumors
4- Radiotherapy and radiosurgical techniques for nervous system tumors
5- Natural history and adjuvant treatments for nervous system tumors
6- Pharmacological and rehabilitative treatments for neurological complications of nervous system tumors
7- Management of the behavioral/psychological problems of the patient and caregiver and end-of-life issues
8- Translational studies on tumors of the nervous system

29) In the context of the multidisciplinary neuro-oncology board, who do you believe the leader of the group should be:
1- the physician with the widest clinical or research experience, regardless of his/her specialty of origin
2- the neurosurgeon
3- the neurologist and/or the clinical neuro-oncologist;
4- the radiation oncologist
5- other physician
6- none: each specialist acts on an equal footing.

PART 4 – PERSPECTIVES
30) Have you ever heard of neuro-oncology mentorship programs?
1- Yes
0- No

31) What should be the role of the mentor in the training of the young neuro-oncologist? Please rank options from the most important (1) to the least important (5).
1- Tutor in the acquisition of specialized technical skills
2- Tutor in the acquisition of relational skills with colleagues and patients
3- Tutor in the acquisition of a method for scientific research, development of a research project and scientific writing, which guarantees the transition towards scientific autonomy
4- Tutor in the acquisition of a rigorous method of peer review
5- Be a model of reference for the elaboration of own career goals and for the definition of an

adequate balance between work and personal life.

32) What should be the role of the AINO Youngster Committee in fostering education of young neuro- oncologist? Please rank options from the most important (1) to the least important (5).
1- Promote the collaboration between different specialists in order to foster the culture of multidisciplinarity
2- Provide a constant platform for the exchange of ideas, useful in clinical practice (e.g., for the management of complex cases) and for the diffusion of information relating to open positions and job opportunities
3- Promote training initiatives (e.g., point out neuro-oncology meeting, disseminate news from the literature, organize webinars and dedicated sessions in the National Congress)
4- Promote scientific research initiatives (cooperative studies, collection of case studies, etc.)
5- Other

33) What could be the role of the society AINO in improving neuro-oncology education in Italy?
1- Promote the inclusion of dedicated neuro-oncology programs in Specialty Schools;
2- Promote neuro-oncology training courses within the single-specialty scientific societies
3- Promote post-graduate neuro-oncology courses;
4- Promote fellowships in the main Italian Neuro-oncology centers
9- Other

34) If you like, please enter a free comment on education in Neuro-Oncology, including aspects not covered in the previous questions, personal experiences, proposals.
