# Supplementary material for: Education paths in neuro-oncology: combining technical skills with multidisciplinary care. A survey from the AINO (Italian Association for Neuro-Oncology) Youngster Committee
Source: J Neurooncol. 2025 Mar 18;173(2):469–77. doi: 10.1007/s11060-025-05003-2 (PMC12106497; doi:10.1007/s11060-025-05003-2)
Supplement: Supplementary file 2 — Supplementary Material 2: Supplementary Tables [file 11060_2025_5003_MOESM2_ESM.pdf]

Education Paths in Neuro-Oncology: combining technical skills with multidisciplinary care.
A survey from the AINO (Italian Association for Neuro-Oncology) Youngster Committee.

Supplementary Tables Q%. Detailed responses to questions. *The number after the letter Q identifies the number of the question.*

Supplementary Table Q0. Number of answers per question

Question	*n*
1	254
2	254
3	254
4	254
5	254
6	254
7	254
8	253
9	254
10	254
11	254
12	254
13	170
14	170
15	224
16	224
17	221
18	224
19	224
20	224
21	221
22	224
23	224
24	224
25	224
26	204
27	204
28	204
29	204
30	196
31	196
32	196
33	196

PART 1 - CHARACTERISTICS OF THE PARTICIPANT

Supplementary Table Q1. Age (years)

**Option**	***n***	**%**
<30	34	13.4
30-39	125	49.2
40-49	63	24.8
50-59	20	7.9
≥ 60	12	4.7
**Total**	**254**	**100**

Supplementary Table Q2. Gender

**Option**	***n***	**%**
Female	123	48.4
Male	127	50.0
I prefer not to specify	4	1.6
**Total**	**254**	**100**

Supplementary Table Q3. Which is your specialty?

**Option**	***n***	**%**
Pathologist	33	13.0
Neurosurgeon	122	48.0
Neurologist	31	12.2
Neuropsychologist	2	0.8
Medical Oncologist	11	4.3
Radiologist	9	3.5
Radiation Oncologist	33	13.0
Other*	13	5.1
**Total**	**254**	**100**

*Pediatrician (n=8), Molecular biologist (n=3), other (n=2).

Supplementary Table Q4. How many years have you been involved in neuro-oncology?

**Option**	***n***	**%**
Less than 5	112	44.1
5–10	69	27.2
11-20	48	18.9
More than 20	25	9.8
**Total**	**254**	**100**

Supplementary Table Q5. What type of organization do you work for?

Option	*n*	%
Non-teaching hospital	81	31.9
University Hospital	79	31.1
Scientific Hospital (IRCCS)	67	26.4
Mixed	24	9.4
Private pratictioner	3	1.2
**Total**	**254**	**100**

Supplementary Table Q6. Where do you work?

Option	*n*	%
North	107	42.1
Centre	79	31.1
South	39	15.4
Islands	23	9.1
Outside of Italy	6	2.4
**Total**	**254**	**100**

Supplementary Table Q7. Where did you carry out your postgraduate education?

Option	*n*	%
North	107	42.1
Centre	97	38.2
South	33	13.0
Islands	16	6.3
Outside of Italy	1	0.4
**Total**	**254**	**100**

Supplementary Table Q7A. According to place of work

Place of Education	Place of Work										Total
	North		Centre		South		Islands		Outside of Italy		
	n	%									
North	96	89.7%	2	2.5%	4	10.3%	3	13.0%	2	33.3%	107
Centre	8	7.5%	73	92.4%	10	25.6%	4	17.4%	2	33.3%	97
South	3	2.8%	2	2.5%	25	64.1%	2	8.7%	1	16.7%	33
Islands	0	0%	2	2.5%	0	0%	14	60.9%	0	0%	16
Outside of Italy	0	%	0	0%	0	0%	0	0%	1	16.7%	1
**Total**	**107**	**100%**	**79**	**100%**	**39**	**100%**	**23**	**100%**	**6**	**100%**	**254**

Supplementary Table Q8. What is your role in the working group?

**Option**	***n***	**%**
Resident	57	22.5
PhD student/ research fellow	18	7.1
Physician	134	53.0
Group Head	9	3.6
Director	12	4.7
Private pratictioner	14	5.5
University Professor	20	7.9
**Total**	**253**	**100**

*More than one role could be selected for this question. One participant gave no responses. Multiple responses were selected in 11 cases (university professor + physician, n=3; university professor + group head, n=1; University professor + director, n=2; resident + PhD student, n=2; PhD student + physician, n=2; physician + group head, n=1).

Supplementary Table Q9. What is the neuro-oncological case load at your Institution, including new diagnoses and follow-up?

**Option**	***n***	**%**
Less than 50 cases	18	7.1
50–100 cases	79	31.1
100–500 cases	103	40.6
More than 500 cases	54	21.3
**Total**	**254**	**100**

Supplementary Table Q10. Does Neuro-oncology Tumor Board routinely operate at your Institution?

**Option**	***n***	**%**
Yes, with weekly or multi-weekly meetings	169	66.5
Yes, with fortnightly meetings	40	15.7
Yes, with monthly meetings	12	4.7
No	33	13.0
**Total**	**254**	**100**

Supplementary Table Q10A. Depending on Institution type

**Option**	Non-teaching hospital		University Hospital		Scientific Hospital (IRCCS)		Mixed	
	n	**%**	***n***	**%**	***n***	**%**	***n***	**%**
Yes, weekly	41	50.6	51	64.6	59	88.1	16	66.7
Yes, fortnightly	16	19.8	15	19.0	4	6.0	4	16.7
Yes, monthly	5	6.2	3	3.8	2	3.0	2	8.3
No	19	23.5	10	12.7	2	3.0	2	8.3
**Total**	**81**	**100**	**79**	**100**	**67**	**100**	**24**	**100**

Supplementary Table Q11. Does your Institution organize educational neuro-oncology meetings?

**Option**	***n***	**%**
Yes, on a regular basis, at least once a month	48	18.9
Yes, on a regular basis, less than once a month	37	14.6
Yes, occasionally	107	42.1
No	62	24.4
**Total**	**254**	**100**

Supplementary Table Q11A. Depending on Institution type

**Option**	Non-teaching hospital		University Hospital		Scientific Hospital (IRCCS)		Mixed	
	n	**%**	***n***	**%**	***n***	**%**	***n***	**%**
Yes, on a regular basis, at least once a month	9	11.1%	15	19.0%	20	29.9%	4	16.7%
Yes, on a regular basis, less than once a month	2	2.5%	12	15.2%	21	31.3%	2	8.3%
Yes, occasionally	39	48.1%	32	40.5%	21	31.3%	12	50.0%
No	31	38.3%	20	25.3%	5	7.5%	6	25.0%
**Total**	**81**	**100%**	**79**	**100%**	**67**	**100%**	**24**	**100%**

Supplementary Table Q12. Are you actively involved in neuro-oncology research?

**Option**	***n***	**%**
Yes	170	66.9
No	84	33.1
**Total**	**254**	**100**

Supplementary Table Q13. If Yes, what kind of research?

**Option**	***n***	**%**
Surgical research	64	37.6
Clinical research	121	71.2
Translational research	79	46.5
**Total**	**170**	**100**

Supplementary Table Q14. Which percentage of your work time do you spend on research activities? Which percentage of your work time do you spend on research activities?

**Option**	***n***	**%**
<10%	43	25.3
10-30%	82	48.2
30-50%	37	21.8
>50%	8	4.7
**Total**	**170**	**100**

PART 2 – NEURO-ONCOLOGY TRAINING AND EDUCATION

Supplementary Table Q15. What is the main reason of your involvement in neuro-oncology?

Option	*n*	%
Spontaneous vocation	138	61.6
Legacy from the mentor	43	19.2
Need of the Istitution you work for	43	19.2
**Total**	**224**	**100**

Supplementary Table Q16. At what stage of your career did you start dealing with neuro-oncology?

Option	*n*	%
Medical Student	48	21.4
Resident	114	50.9
PhD student/research fellow	18	8.0
After job placement	44	19.6
**Total**	**224**	**100**

Supplementary Table Q17. What were the most important steps in your neuro-oncology education?
Please rank options from the most important to the least important.

Option	Rank 1		Rank 2		Rank 3		Rank 4		Total
	n	%	*n*	%	*n*	%	*n*	%	
Residency Program	46	32.6	27	22.7	43	26.2	100	40.3	**216**
Ph.D. Program	39	27.7	16	13.4	20	12.2	36	14.5	**111**
Fellowships in Italy or abroad	40	28.4	28	23.5	30	18.3	45	18.1	**143**
Conferences and courses	16	11.3	48	40.3	71	43.3	67	27.0	**203**
**Total***	**141**	**100**	**119**	**100**	**164**	**100**	**248**	**100**	**221**

* Responders: 221. In questions involving ranking of multiple options (questions no. 17, 28, 31 and 32) we found several inconsistent answers, including missing rank or same rank given to different options. The elaboration of data reflects such inconsistencies.

Supplementary Table Q18. Do you believe that the Residency Program you attended adequately prepared you for your neuro-oncology activity?

Option	*n*	%
Yes, in a complete and exhaustive way	56	25.0
Sufficiently	92	41.1
Scarcely	45	20.1
Not at all	31	13.8
**Total**	**224**	**100**

Supplementary Table Q18A. Divided by age

Option	Age <30		Age 30-39		Age 40-49		Age 50-59		Age >60	
	n	%	*n*	%	*n*	%	*n*	%	*n*	%
Yes, in a complete and exhaustive way	7	25.0	34	30.4	11	20.0	2	11.1	2	18.2
Sufficiently	10	35.7	50	44.6	20	36.4	10	55.6	2	18.2
Scarcely	11	39.3	19	17.0	7	12.7	3	16.7	5	45.5
Not at all	0	0.0	9	8.0	17	30.9	3	16.7	2	18.2
**Total**	**28**	**100**	**112**	**100**	**55**	**100**	**18**	**100**	**11**	**100**

Supplementary Table Q19. What were the strength points of your Residency Program in the neuro-oncology field?

**Option**	***n***	**%**
Technical and scientific preparation to deal with neuro-oncological diseases	165	73.7
Preparation to manage the psychological stress and care load caused by the neuro-oncological patient	79	35.3
Preparation to manage multidisciplinary neuro-oncological interaction	116	51.8
Other	11	4.9
**Total***	**224**	**100**

*This was a multiple-choice multiple-answer question. The number of responders was 224.

Supplementary Table Q20. What were the weaknesses and/or aspects to be improved of your Residency Program in the neuro-oncology field?

**Option**	***n***	**%**
Insufficient technical and scientific preparation to deal with neuro-oncological diseases	81	36.2
Insufficient preparation to manage the psychological stress and care load caused by the neuro-oncological patient	77	34.4
Insufficient preparation to manage multidisciplinary neuro-oncological interaction	70	31.3
Other	35	15.6
**Total***	**224**	**100**

*This was a multiple-choice multiple-answer question. The number of responders was 224.

Supplementary Table Q21. If you have attended a PhD Program, do you think it has prepared you for clinical neuro-oncology activity?

**Option**	***n***	**%**
Very much	14	18.9
Sufficiently	12	16.2
Scarcely	26	35.1
No: the PhD Program only prepared me for neuro-oncology research	22	29.7
**Total***	74	100
I did not attend a PhD Program	147	NA

* Total answers: 221. NA, not applicable

Supplementary Table Q22. What is your main tool for Continuing Neuro-Oncology Education?

**Option**	***n***	**%**
National Meetings	27	12.1
International Meetings	18	8.0
Masters/scholarships/ courses	7	3.1
Tumor board	26	11.6
Scientific literature	123	54.9
Daily clinical practice	23	10.3
**Total**	**224**	**100**

Supplementary Table Q22A. Divided by age

**Option**	**Age <30**		**Age 30-39**		**Age 40-49**		**Age 50-59**		**Age >60**	
	n	**%**	***n***	**%**	***n***	**%**	***n***	**%**	***n***	**%**
National Meetings	3	10.7	13	11.6	10	18.2	1	5.6	0	0.0
International Meetings	0	0.0	12	10.7	3	5.5	3	16.7	0	0.0
Masters/scholarships/ courses	3	10.7	2	1.8	1	1.8	0	0.0	1	9.1
Tumor board	4	14.3	8	7.1	10	18.2	4	22.2	0	0.0
Scientific literature	13	46.4	62	55.4	30	54.5	8	44.4	10	90.9
Daily clinical practice	5	17.9	15	13.4	1	1.8	2	11.1	0	0.0
**Total**	**28**	**100**	**112**	**100**	**55**	**100**	**18**	**100**	**11**	**100**

Supplementary Table Q22B. *Divided by Structure*

**Option**	Non-teaching hospital		University Hospital		Scientific Hospital (IRCCS)		Mixed	
	n	**%**	***n***	**%**	***n***	**%**	***n***	**%**
National Meetings	9	13.2	8	11.3	7	11.5	3	13.6
International Meetings	4	5.9	5	7.0	6	9.8	3	13.6
Masters/scholarships/ courses	2	2.9	3	4.2	1	1.6	1	4.5
Tumor board	8	11.8	8	11.3	9	14.8	1	4.5
Scientific literature	37	54.4	43	60.6	28	45.9	13	59.1
Daily clinical practice	8	11.8	4	5.6	10	16.4	1	4.5
**Total**	**68**	**100**	**71**	**100**	**61**	**100**	**22**	**100**

Supplementary Table Q23. Do your Residency or Ph.D. Programs include a period abroad to deepen the knowledge of neuro-oncological topics of interest?

**Option**	***n***	**%**
Yes, but short-lived (<3 months)	17	7.6
Yes, up to 6 months	40	17.9
Yes, from 6 to 12 months	47	21.0
Yes, more than 1-year long	33	14.7
No	87	38.8
**Total**	**224**	**100**

Supplementary Table Q23A. Divided by age

**Option**	**Age <30**		**Age 30-39**		**Age 40-49**		**Age 50-59**		**Age >60**	
	n	**%**	***n***	**%**	***n***	**%**	***n***	**%**	***n***	**%**
Yes, but short-lived (<3 months)	3	10.7	5	4.5	3	5.5	3	16.7	3	27.3
Yes, up to 6 months	6	21.4	21	18.8	8	14.5	4	22.2	1	9.1
Yes, from 6 to 12 months	6	21.4	29	25.9	10	18.2	1	5.6	1	9.1
Yes, more than 1-year long	7	25.0	21	18.8	5	9.1	0	0.0	0	0.0
No	6	21.4	36	32.1	29	52.7	10	55.6	6	54.5
**Total**	**28**	**100**	**112**	**100**	**55**	**100**	**18**	**100**	**11**	**100**

Supplementary Table Q24. During your training, did you have experience in basic/translational research?

**Option**	***n***	**%**
Yes	125	55.8
No	99	44.2
I would have been interested in	77	77.8
I would not have been interested in	22	22.2
**Total**	**224**	**100**

Supplementary Table Q25. During your training, have you participated in clinical trials as subinvestigator or study coordinator?

**Option**	***n***	**%**
Yes	87	38.8
No	137	61.2
I would have been interested in	113	82.5
I would not have been interested in	24	17.5
**Total**	**224**	**100**

PART 3 – YOUR VISION OF NEURO-ONCOLOGY

Supplementary Table Q26. What are the ideal modalities and frequency of Continuous Neuro-oncology Education, in your opinion?

Option	*n*	%
Participation in at least one neuro-oncology meeting/course per year	62	30.4
Participation in more than one neuro-oncology meeting/course per year	121	59.3
It is sufficient to keep up to date with the guidelines and literature	20	9.8
I do not think a particular continuous education schedule is necessary	1	0.5
**Total**	**204**	**100**

Supplementary Table Q26A. Divided by age

**Option**	**Age <30**		**Age 30-39**		**Age 40-49**		**Age 50-59**		**Age >60**	
	n	**%**	***n***	**%**	***n***	**%**	***n***	**%**	***n***	**%**
Participation in at least one neuro-oncology meeting/course per year	6	26,1%	31	29,8%	14	28,0%	8	47,1%	3	30,0%
Participation in more than one neuro-oncology meeting/course per year	16	69,6%	62	59,6%	30	60,0%	7	41,2%	6	60,0%
It is sufficient to keep up to date with the guidelines and literature	1	4,3%	10	9,6%	6	12,0%	2	11,8%	1	10,0%
I do not think a particular continuous education schedule is necessary	0	0%	1	1,0%	0	0%	0	0%	0	0%
**Total**	**23**	**100**	**104**	**100**	**50**	**100**	**17**	**100**	**10**	**100**

Supplementary Table Q26B. Divided by Structure

**Option**	Non-teaching hospital		University Hospital		Scientific Hospital (IRCCS)		Mixed	
	n	**%**	***n***	**%**	***n***	**%**	***n***	**%**
Participation in at least one neuro-oncology meeting/course per year	18	29,0%	17	26,2%	16	28,6%	11	55,0%
Participation in more than one neuro-oncology meeting/course per year	37	59,7%	41	63,1%	33	58,9%	9	45,0%
It is sufficient to keep up to date with the guidelines and literature	6	9,7%	7	10,8%	7	12,5%	0	0%
I do not think a particular continuous education schedule is necessary	1	1,6%	0	0%	0	0%	0	0%
**Total**	**62**	**100**	**65**	**100**	**56**	**100**	**20**	**100**

Supplementary Table Q26C. Divided by Specialty

Option	Pathologist		Neurosurgeon		Neurologist		Neuropsychologist		Medical Oncologist		Radiologist		Radiation Oncologist		Other	
	n	%	n	%	n	%	n	%	n	%	n	%	n	%	n	%
Participation in at least one neuro-oncology meeting/course per year	9	34.6%	27	28.7%	10	34.5%	1	50.0%	4	50.0%	2	28.6%	7	26.9%	2	16.7%
Participation in more than one neuro-oncology meeting/course per year	16	61.5%	51	54.3%	17	58.6%	1	50.0%	4	50.0%	4	57.1%	18	69.2%	10	83.3%
It is sufficient to keep up to date with the guidelines and literature	1	3.9%	15	16.0%	2	6.9%	0	0%	0	0%	1	14.3%	1	3.8%	0	0%
I do not think a particular continuous education schedule is necessary	0	0%	1	1.1%	0	0%	0	0%	0	0%	0	00%	0	0%	0	0%
**Total**	**26**	**100%**	**94**	**100%**	**29**	**100%**	**2**	**100%**	**8**	**100%**	**7**	**100.00%**	**26**	**100%**	**12**	**100%**

Supplementary Table Q27. The multidisciplinary collaboration in neuro-oncology is, in your opinion:

Option	*n*	%
Fundamental: care directives must always come from the multidisciplinary group	186	91.2
Useful, but the referring specialist retains the direction of care of his patient	17	8.3
Optional, as each specialist must do his or her work independently of the others	1	0.5
**Total**	**204**	**100**

Supplementary Table Q27A. Divided by age

Option	Age <30		Age 30-39		Age 40-49		Age 50-59		Age >60	
	n	%	*n*	%	*n*	%	*n*	%	*n*	%
Fundamental: care directives must always come from the multidisciplinary group	22	95.7%	96	92.3%	47	94.0%	14	82.4%	7	70.0%
Useful, but the referring specialist retains the direction of care of his patient	1	4.3%	7	6.7%	3	6.0%	3	17.6%	3	30.0%
Optional, as each specialist must do his or her work independently of the others	0	0%	1	0.96%	0	0%	0	0%	0	0%
**Total**	**23**	**100**	**104**	**100**	**50**	**100**	**17**	**100**	**10**	**100**

Supplementary Table Q27B. Divided by Structure

Option	Non-teaching hospital		University Hospital		Scientific Hospital (IRCCS)		Mixed	
	n	**%**	***n***	**%**	***n***	**%**	***n***	**%**
Fundamental: care directives must always come from the multidisciplinary group	54	87.1%	57	87.7%	56	100.0%	18	90.0%
Useful, but the referring specialist retains the direction of care of his patient	7	11.3%	8	12.3%	0	0%	2	10.0%
Optional, as each specialist must do his or her work independently of the others	1	1.6%	0	0%	0	0%	0	0%
**Total**	**62**	**100**	**65**	**100**	**56**	**100**	**20**	**100**

Supplementary Table Q27C. Divided by Specialty

**Option**	**Pathologist**		**Neurosurgeon**		**Neurologist**		**Neuropsychologist**		**Medical Oncologist**		**Radiologist**		**Radiation Oncologist**		**Other**	
	**n**	**%**	**n**	**%**	**n**	**%**	**n**	**%**	**n**	**%**	**n**	**%**	**n**	**%**	**n**	**%**
Fundamental: care directives must always come from the multidisciplinary group	21	80.8 %	83	88.3 %	27	93.1 %	2	100 %	8	100 %	7	100 %	26	100 %	12	100 %
Useful, but the referring specialist retains the direction of care of his patient	4	15.4 %	11	11.7 %	2	6.9 %	0	0%	0	0%	0	0%	0	0%	0	0%
Optional, as each specialist must do his or her work independently of the others	1	3.8%	0	0%	0	0%	0	0%	0	0%	0	0%	0	0%	0	0%
**Total**	**26**	**100 %**	**94**	**100 %**	**29**	**100 %**	**2**	**100 %**	**8**	**100 %**	**7**	**100 %**	**26**	**100 %**	**12**	**100 %**

Supplementary Table Q28. The multidisciplinary neuro-oncology collaboration requires that each specialist gains a certain degree of knowledge of technical aspects concerning specializations other than his/hers. Please rank the following skills according to what you think are the most important to define yourself as a neuro-oncology care provider (1: most important; 8: least important; 9:N/A)
Please rank the following skills according to what you think are the most important to define yourself as a neuro-oncology care provider (excluding your own skills)

Option	Rank 1		Rank 2		Rank 3		Rank 4		Rank 5		Rank 6		Rank 7		Rank 8		Tot.
	n	%	*n*	%	*n*	%	*n*	%	*n*	%	*n*	%	*n*	%	*n*	%	
Diagnostic imaging of nervous system tumors	5	10.4%	13	21.3%	6	8.2%	8	6.6	9	4.5%	23	9.7	46	13.3%	93	17.9%	203
Surgical techniques for nervous system tumors resection	7	14.6%	8	13.1%	14	19.2%	19	15.7	34	17.1%	18	7.6	35	10.1%	61	11.7%	196
Pathological/molecular features of nervous system tumors	10	20.8%	9	14.8%	4	5.5%	9	7.4	16	8.0%	19	8.0	40	11.6%	95	18.2%	202
Radiotherapy and radiosurgical techniques of nervous system tumors	1	2.1%	5	8.2%	9	12.3%	24	19.8	29	14.6%	44	18.5	42	12.2%	44	8.4%	198
Natural history and adjuvant treatments of nervous system tumors	6	12.5%	8	13.1%	7	9.6%	12	9.9	17	8.5%	26	10.9	45	13.0%	80	15.4%	201
Pharmacological and rehabilitative treatments for neurological complications of nervous system tumors	2	4.2%	7	11.5%	7	9.6%	19	15.7	33	16.6%	39	16.4	40	11.6%	56	10.7%	203
Management of the behavioral/psychological problems of the patient and caregiver and end-of-life issues	3	6.3%	3	4.9%	19	26.0%	12	9.9	36	18.1%	38	16.0	48	13.9%	44	8.4%	203
Translational studies on tumors of the nervous system	14	29.2%	8	13.1%	7	9.6%	18	14.9	25	12.6%	31	13.0	49	14.2%	48	9.2%	200
**Total***	**48**	**100%**	**61**	**100%**	**73**	**100%**	**121**	**100%**	**199**	**100%**	**238**	**100%**	**345**	**100%**	**521**	**100%**	**204**

* Responders: 204. In questions involving ranking of multiple options (questions no. 17, 28, 31 and 32) we found several inconsistent answers, including missing rank or same rank given to different options. The elaboration of data reflects such inconsistencies.

Supplementary Table Q28A. Options ranked 1-3

**Option**	**Rank 1-3**	
	n	**%***
Diagnostic imaging of nervous system tumors	24	11.8%
Surgical techniques for nervous system tumors resection	29	14.2%
Pathological/molecular features of nervous system tumors	23	11.3%
Radiotherapy and radiosurgical techniques of nervous system tumors	15	7.4%
Natural history and adjuvant treatments of nervous system tumors	21	10.3%
Pharmacological and rehabilitative treatments for neurological complications of nervous system tumors	16	7.8%
Management of the behavioral/psychological problems of the patient and caregiver and end-of-life issues	25	12.3%
Translational studies on tumors of the nervous system	29	14.2%

*Of 204 total responders

Supplementary Table Q28B. Options ranked 1-3 divided by age

**Option Ranked 1-3**	**Age <30**		**Age 30-39**		**Age 40-49**		**Age 50-59**		**Age >60**	
	n	**%**	***n***	**%**	***n***	**%**	***n***	**%**	***n***	**%**
Diagnostic imaging of nervous system tumors	1	4.3%	11	10.6%	11	22%	1	5.9%	0	0
Surgical techniques for nervous system tumors resection	2	8.7%	17	16.3%	8	16%	2	11.8%	0	0
Pathological/molecular features of nervous system tumors	0	0	10	9.6%	11	22%	1	5.9%	1	10%
Radiotherapy and radiosurgical techniques of nervous system tumors	0	0	10	9.6%	3	6%	1	5.9%	1	10%
Natural history and adjuvant treatments of nervous system tumors	0	0	9	8.7%	10	20%	1	5.9%	1	10%
Pharmacological and rehabilitative treatments for neurological complications of nervous system tumors	1	4.3%	7	6.7%	5	10%	2	11.8%	1	10%
Management of the behavioral/psychological problems of the patient and caregiver and end-of-life issues	2	8.7%	13	12.5%	5	10%	3	17.6%	2	20%
Translational studies on tumors of the nervous system	1	4.3%	12	11.5%	7	14%	8	47.1%	1	10%
**Total***	**23**	**NA**	**104**	**NA**	**50**	**NA**	**17**	**NA**	**10**	**NA**

*Of 204 responders according to age

Supplementary Table Q28C. Options ranked 1-3 divided by Structure

**Option**	Non-teaching hospital		University Hospital		Scientific Hospital (IRCCS)		Mixed	
	n	**%**	***n***	**%**	***n***	**%**	***n***	**%**
Diagnostic imaging of nervous system tumors	12	19.4%	5	7.7%	5	8.9%	2	10.0%
Surgical techniques for nervous system tumors resection	13	21.0%	7	10.8%	6	10.7%	3	15.0%
Pathological/molecular features of nervous system tumors	11	17.7%	4	6.2%	6	10.7%	2	10.0%
Radiotherapy and radiosurgical techniques of nervous system tumors	6	9.7%	3	4.6%	3	5.4%	3	15.0%
Natural history and adjuvant treatments of nervous system tumors	12	19.4%	1	1.5%	5	8.9%	2	10.0%
Pharmacological and rehabilitative treatments for neurological complications of nervous system tumors	7	11.3%	8	12.3%	0	0.0%	1	5.0%
Management of the behavioral/psychological problems of the patient and caregiver and end-of-life issues	10	16.1%	11	16.9%	3	5.4%	1	5.0%
Translational studies on tumors of the nervous system	10	16.1%	13	20.0%	4	7.1%	2	10.0%
**Total***	**62**	**NA**	**65**	**NA**	**56**	**NA**	**20**	**NA**

*Of 204 responders according to structure type

Supplementary Table Q28D. Options ranked 1-3 divided by Specialty

Option	Pathologist		Neurosurgeon		Neurologist		Neuropsychologist		Medical Oncologist		Radiologist		Radiation Oncologist		Other	
	**n**	**%**	**n**	**%**	**n**	**%**	**n**	**%**	**n**	**%**	**n**	**%**	**n**	**%**	**n**	**%**
Diagnostic imaging of nervous system tumors	3	11.5 %	11	11.7 %	2	6.9 %	0	0%	0	0%	1	14.3 %	6	23.1 %	1	8.3 %
Surgical techniques for nervous system tumors resection	5	19.2 %	11	11.7 %	5	17.2 %	1	50%	1	12.5 %	1	14.3 %	5	19.2 %	0	0%
Pathological/molecular features of nervous system tumors	3	11.5 %	9	9.6 %	2	6.9 %	0	0%	0	0%	1	14.3 %	7	26.9 %	1	8.3 %
Radiotherapy and radiosurgical techniques of nervous system tumors	1	3.8%	6	6.4 %	3	10.3 %	0	0%	0	0%	1	14.3 %	3	11.5 %	1	8.3 %
Natural history and adjuvant treatments of nervous system tumors	3	11.5 %	9	9.6 %	2	6.9 %	0	0%	0	0%	1	14.3 %	5	19.2 %	1	8.3 %
Pharmacological and rehabilitative treatments for neurological complications of nervous system tumors	4	15.4 %	8	8.5 %	1	3.4 %	0	0%	0	0%	1	14.3 %	2	7.7 %	0	0%
Management of the behavioral/psychological problems of the patient and caregiver and end-of-life issues	8	30.8 %	12	12.8 %	3	10.3 %	0	0%	0	0%	0	0	2	7.7 %	0	0%
Translational studies on tumors of the nervous system	3	11.5 %	16	17.0 %	4	13.8 %	0	0%	0	0%	1	14.3 %	4	15.4 %	1	8.3 %
**Total***	**26**	**NA**	**94**	**NA**	**29**	**NA**	**2**	**NA**	**8**	**NA**	**7**	**NA**	**26**	**NA**	**12**	**NA**

*Of 204 responders according to specialty

Supplementary Table Q29. In the context of the multidisciplinary neuro-oncology board, who do you believe the leader of the group should be:

Option	*n*	%
The physician with the widest clinical or research experience, regardless of his/her specialty of origin	90	44.1%
The neurosurgeon	11	5.4%
The neurologist and/or the clinical neuro-oncologist;	36	17.6%
The radiation oncologist	0	0%
Other physician	2	1.0%
None: each specialist acts on an equal footing	65	31.9%
**Total**	**204**	**100**

Supplementary Table Q29A. Divided by age

Option	Age <30		Age 30-39		Age 40-49		Age 50-59		Age >60	
	n	%	*n*	%	*n*	%	*n*	%	*n*	%
The physician with the widest clinical or research experience, regardless of his/her specialty of origin	12	52.2%	49	47.1%	19	38.0%	7	41.2%	3	30%
The neurosurgeon	2	8.7%	3	2.9%	3	6.0%	2	11.8%	1	10%
The neurologist and/or the clinical neuro-oncologist;	5	21.7%	17	16.3%	10	20.0%	2	11.8%	2	20%
The radiation oncologist	0	0%	0	0%	0	0%	0	0%	0	0%
Other physician	0	0.0%	1	1.0%	1	2.0%	0	0.0%	0	0.0%
None: each specialist acts on an equal footing	4	17.4%	34	32.7%	17	34.0%	6	35.3%	4	40%
**Total**	**23**	**100.0 %**	**104**	**100.0 %**	**50**	**100.0 %**	**17**	**100.0 %**	**10**	**100.0 %**

Supplementary Table Q29B. Divided by Structure

Option	Non-teaching hospital		University Hospital		Scientific Hospital (IRCCS)		Mixed	
	n	%	*n*	%	*n*	%	*n*	%
The physician with the widest clinical or research experience, regardless of his/her specialty of origin	27	43.5%	32	49.2%	21	37.5%	10	50.0%
The neurosurgeon	4	6.5%	7	10.8%	0	0%	0	0%
The neurologist and/or the clinical neuro-oncologist;	8	12.9%	10	15.4%	15	26.8%	2	10.0%
The radiation oncologist	0	0%	0	0%	0	0%	0	0%
Other physician	1	1.6%	0	0%	1	1.8%	0	0%
None: each specialist acts on an equal footing	22	35.5%	16	24.6%	19	33.9%	8	40.0%
**Total**	**62**	**100%**	**65**	**100%**	**56**	**100%**	**20**	**100%**

Supplementary Table Q29C. Divided by Specialty

**Option**	**Pathologist**		**Neurosurgeon**		**Neurologist**		**Neuropsychologist**		**Medical Oncologist**		**Radiologist**		**Radiation Oncologist**		**Other**	
	**n**	**%**	**n**	**%**	**n**	**%**	**n**	**%**	**n**	**%**	**n**	**%**	**n**	**%**	**n**	**%**
The physician with the widest clinical or research experience, regardless of his/her specialty of origin	13	50.0%	47	50.0%	9	31.0%	0	0%	4	50.0%	2	28.6%	9	34.6%	6	50.0%
The neurosurgeon	0	0%	11	11.7%	0	0%	0	0%	0	0%	0	0%	0	0%	0	0%
The neurologist and/or the clinical neuro-oncologist;	7	26.9%	6	6.4%	11	37.9%	1	50.0%	3	37.5%	1	14.3%	3	11.5%	4	33.3%
The radiation oncologist	0	0%	0	0%	0	0%	0	0%	0	0%	0	0%	0	0%	0	0%
Other physician	0	0%	1	1.1%	1	3.4%	0	0%	0	0%	0	0%	0	0%	0	0%
None: each specialist acts on an equal footing	6	23.1%	29	30.9%	8	27.6%	1	50.0%	1	12.5%	4	57.1%	14	53.8%	2	16.7%
**Total**	**26**	**100%**	**94**	**100%**	**29**	**100%**	**2**	**100%**	**8**	**100%**	**7**	**100%**	**26**	**100%**	**12**	**100%**

PART 4 – PERSPECTIVES

Supplementary Table Q30. Have you ever heard of neuro-oncology mentorship programs?

**Option**	***n***	**%**
Yes	78	39.8%
No	118	60.2%
**Total**	**196**	**100%**

Supplementary Table Q30A. Divided by age

**Option**	**Age <30**		**Age 30-39**		**Age 40-49**		**Age 50-59**		**Age >60**	
	n	**%**	***n***	**%**	***n***	**%**	***n***	**%**	***n***	**%**
Yes	4	18.2%	46	45.5%	19	39.6%	6	35.3%	3	37.5%
No	18	81.8%	55	54.5%	29	60.4%	11	64.7%	5	62.5%
**Total**	**22**	**100%**	**101**	**100%**	**48**	**100%**	**17**	**100%**	**8**	**100%**

Supplementary Table Q30B. Divided by Structure

**Option**	Non-teaching hospital		University Hospital		Scientific Hospital (IRCCS)		Mixed	
	n	**%**	***n***	**%**	***n***	**%**	***n***	**%**
Yes	14	23.3%	30	47.6%	26	48.1%	7	38.9%
No	46	76.7%	33	52.4%	28	51.9%	11	61.1%
**Total**	**60**	**100%**	**63**	**100%**	**54**	**100%**	**18**	**100%**

Supplementary Table Q31. What should be the role of the mentor in the training of the young neuro-oncologist? *Please rank options from the most important to the least important.*

**Option**	**Rank 1**		**Rank 2**		**Rank 3**		**Rank 4**		**Rank 5**		**Tot.**
	n	**%**	***n***	**%**	***n***	**%**	***n***	**%**	***n***	**%**	
Tutor in the acquisition of specialized technical skills	37	21.1%	11	6.2%	11	6.0%	26	13.5%	98	51.6%	**183**
Tutor in the acquisition of relational skills with colleagues and patients	16	9.1%	43	24.3%	54	29.3%	52	27.1%	11	5.8%	**176**
Tutor in the acquisition of a method for scientific research, development of a research project and scientific writing, which guarantees the transition towards scientific autonomy	13	7.4%	39	22.0%	76	41.3%	38	19.8%	16	8.4%	**182**
Tutor in the acquisition of a rigorous method of peer review	46	26.3%	63	35.6%	17	9.2%	40	20.8%	19	10.0%	**185**
Be a model of reference for the elaboration of own career goals and for the definition of an adequate balance between work and personal life	63	36.0%	21	11.9%	26	14.1%	36	18.8%	46	24.2%	**192**
**Total***	**175**	**100%**	**177**	**100%**	**184**	**100%**	**192**	**100%**	**190**	**100%**	**196**

* Responders: 196. In questions involving ranking of multiple options (questions no. 17, 28, 31 and 32) we found several inconsistent answers, including missing rank or same rank given to different options. The elaboration of data reflects such inconsistencies.

Supplementary Table Q31A. Options ranked 1-3

**Option**	**Rank 1-2**	
	n	**%***
Tutor in the acquisition of specialized technical skills	48	24.5%
Tutor in the acquisition of relational skills with colleagues and patients	59	30.1%
Tutor in the acquisition of a method for scientific research, development of a research project and scientific writing, which guarantees the transition towards scientific autonomy	52	26.5%
Tutor in the acquisition of a rigorous method of peer review	109	55.6%
Be a model of reference for the elaboration of own career goals and for the definition of an adequate balance between work and personal life	84	42.9%

*Of 196 total responders

Supplementary Table Q31B. Options ranked 1-3 divided by age

**Option Ranked 1-2**	**Age <30**		**Age 30-39**		**Age 40-49**		**Age 50-59**		**Age >60**	
	n	**%**	***n***	**%**	***n***	**%**	***n***	**%**	***n***	**%**
Tutor in the acquisition of specialized technical skills	5	22.7%	18	17.8%	10	20.8%	3	17.6%	4	50.0%
Tutor in the acquisition of relational skills with colleagues and patients	5	22.7%	36	35.6%	12	25.0%	4	23.5%	2	25.0%
Tutor in the acquisition of a method for scientific research, development of a research project and scientific writing, which guarantees the transition towards scientific autonomy	4	18.2%	30	29.7%	13	27.1%	3	17.6%	2	25.0%
Tutor in the acquisition of a rigorous method of peer review	12	54.5%	55	54.5%	26	54.2%	12	70.6%	4	50.0%
Be a model of reference for the elaboration of own career goals and for the definition of an adequate balance between work and personal life	11	50.0%	42	41.6%	21	43.8%	8	47.1%	2	25.0%
**Total***	**22**	**NA**	**101**	**NA**	**48**	**NA**	**17**	**NA**	**8**	**NA**

*Of 196 responders divided by age

Supplementary Table Q31C. Options ranked 1-3 divided by Structure

**Option**	Non-teaching hospital		University Hospital		Scientific Hospital (IRCCS)		Mixed	
	n	**%**	***n***	**%**	***n***	**%**	***n***	**%**
Tutor in the acquisition of specialized technical skills	19	31.7%	14	22.2%	12	22.2%	2	11.1%
Tutor in the acquisition of relational skills with colleagues and patients	18	30.0%	16	25.4%	20	37.0%	4	22.2%
Tutor in the acquisition of a method for scientific research, development of a research project and scientific writing, which guarantees the transition towards scientific autonomy	14	23.3%	17	27.0%	14	25.9%	7	38.9%
Tutor in the acquisition of a rigorous method of peer review	28	46.7%	39	61.9%	31	57.4%	11	61.1%
Be a model of reference for the elaboration of own career goals and for the definition of an adequate balance between work and personal life	27	45.0%	28	44.4%	21	38.9%	8	44.4%
**Total***	**60**	**NA**	**63**	**NA**	**54**	**NA**	**18**	**NA**

*Of 196 responders divided by structure

Supplementary Table Q31D. Options ranked 1-3 divided by Specialty

**Option**	**Pathologist**		**Neurosurgeon**		**Neurologist**		**Neuropsychologist**		**Medical Oncologist**		**Radiologist**		**Radiation Oncologist**		**Other**	
	**n**	**%**	**n**	**%**	**n**	**%**	**n**	**%**	**n**	**%**	**n**	**%**	**n**	**%**	**n**	**%**
Tutor in the acquisition of specialized technical skills	5	20.8%	23	24.7%	7	25.9%	0	0%	1	12.5%	1	14.3%	8	33.3%	3	25.0%
Tutor in the acquisition of relational skills with colleagues and patients	8	33.3%	28	30.1%	9	33.3%	0	0%	2	25.0%	1	14.3%	7	29.2%	4	33.3%
Tutor in the acquisition of a method for scientific research, development of a research project and scientific writing, which guarantees the transition towards scientific autonomy	4	16.7%	24	25.8%	6	22.2%	0	0%	2	25.0%	3	42.9%	8	33.3%	0	0%
Tutor in the acquisition of a rigorous method of peer review	16	66.7%	48	51.6%	14	51.9%	1	100%	6	75.0%	5	71.4%	12	50.0%	0	0%
Be a model of reference for the elaboration of own career goals and for the definition of an adequate balance between work and personal life	13	54.2%	41	44.1%	9	33.3%	1	100%	5	62.5%	2	28.6%	11	45.8%	0	0%
**Total***	**24**	**NA**	**93**	**NA**	**27**	**NA**	**1**	**NA**	**8**	**NA**	**7**	**NA**	**24**	**NA**	**12**	**NA**

*Of 196 responders divided by specialty

Supplementary Table Q32. What should be the role of the AINO Youngster Committee in fostering education of young neuro-oncologist? *Please rank options from the most important to the least important.*

Option	Rank 1		Rank 2		Rank 3		Rank 4		Rank 5		Tot.
	n	%	*n*	%	*n*	%	*n*	%	*n*	%	
Promote the collaboration between different specialists in order to foster the culture of multidisciplinarity	37	34.6 %	31	17.9 %	25	13.7 %	39	20.9 %	51	34.7 %	**183**
Provide a constant platform for the exchange of ideas, useful in clinical practice (e.g., for the management of complex cases) and for the dissemination of information relating to open positions and job opportunities	12	11.2 %	41	23.7 %	49	26.8 %	61	32.6 %	19	12.9 %	**182**
Promote training initiatives (e.g., point out neuro-oncology meeting, disseminate news from the literature, organize webinars and dedicated sessions in the National Congress)	12	11.2 %	30	17.3 %	75	41.0 %	40	21.4 %	25	17.0 %	**182**
Promote scientific research initiatives (cooperative studies, collection of case studies, etc.)	14	13.1 %	70	40.5 %	31	16.9 %	43	23.0 %	28	19.0 %	**186**
Other§	32	29.9 %	1	0.6 %	3	1.6 %	4	2.1 %	24	16.3 %	**64**
**Total**	**107**	**100 %**	**173**	**100 %**	**183**	**100 %**	**187**	**100 %**	**147**	**100 %**	**196**

* Responders: 196. In questions involving ranking of multiple options (questions no. 17, 28, 31 and 32) we found several inconsistent answers, including missing rank or same rank given to different options. The elaboration of data reflects such inconsistencies.

§Other (summary of free text suggestions):

- deal with palliative care
- improve integration among colleagues working in different Institutions
- improve youngsters engagement
- promote national/international fellowships combining surgical, clinical and translational work
- promote working groups dedicated to specific histotypes
- promote interaction between pediatric and adult neuro-oncology
- promote neuro-oncologic paths in Radiation Oncology specialty schools
- suggest topics of interest for AINO meetings

Supplementary Table Q32A. Options ranked 1-3

**Option**	**Rank 1-2**	
	n	**%***
Promote the collaboration between different specialists in order to foster the culture of multidisciplinarity	68	34.7%
Provide a constant platform for the exchange of ideas, useful in clinical practice (e.g., for the management of complex cases) and for the dissemination of information relating to open positions and job opportunities	53	27.0%
Promote training initiatives (e.g., point out neuro-oncology meeting, disseminate news from the literature, organize webinars and dedicated sessions in the National Congress)	42	21.4%
Promote scientific research initiatives (cooperative studies, collection of case studies, etc.)	84	42.9%
Other	33	16.8%

*Of 196 total responders

Supplementary Table Q32B. Options ranked 1-3 divided by age

**Option Ranked 1-2**	**Age <30**		**Age 30-39**		**Age 40-49**		**Age 50-59**		**Age >60**	
	n	**%**	***n***	**%**	***n***	**%**	***n***	**%**	***n***	**%**
Promote the collaboration between different specialists in order to foster the culture of multidisciplinarity	7	31.8%	40	39.6%	13	27.1%	5	29.4%	3	37.5%
Provide a constant platform for the exchange of ideas, useful in clinical practice (e.g., for the management of complex cases) and for the dissemination of information relating to open positions and job opportunities	5	22.7%	24	23.8%	16	33.3%	4	23.5%	4	50.0%
Promote training initiatives (e.g., point out neuro-oncology meeting, disseminate news from the literature, organize webinars and dedicated sessions in the National Congress)	3	13.6%	23	22.8%	12	25.0%	1	5.9%	3	37.5%
Promote scientific research initiatives (cooperative studies, collection of case studies, etc.)	10	45.5%	41	40.6%	22	45.8%	9	52.9%	2	25.0%
Other	4	18.2%	12	11.9%	12	25.0%	4	23.5%	1	12.5%
**Total***	**22**	**NA**	**101**	**NA**	**48**	**NA**	**17**	**NA**	**8**	**NA**

*Of 196 responders divided by age

Supplementary Table Q32C. Options ranked 1-3 divided by Structure

**Option**	Non-teaching hospital		University Hospital		Scientific Hospital (IRCCS)		Mixed	
	n	**%**	***n***	**%**	***n***	**%**	***n***	**%**
Promote the collaboration between different specialists in order to foster the culture of multidisciplinarity	22	36.7%	24	38.1%	17	31.5%	4	22.2%
Provide a constant platform for the exchange of ideas, useful in clinical practice (e.g., for the management of complex cases) and for the dissemination of information relating to open positions and job opportunities	18	30.0%	11	17.5%	15	27.8%	9	50.0%
Promote training initiatives (e.g., point out neuro-oncology meeting, disseminate news from the literature, organize webinars and dedicated sessions in the National Congress)	16	26.7%	11	17.5%	11	20.4%	3	16.7%
Promote scientific research initiatives (cooperative studies, collection of case studies, etc.)	22	36.7%	28	44.4%	26	48.1%	8	44.4%
Other	7	11.7%	13	20.6%	10	18.5%	3	16.7%
**Total***	**60**	**NA**	**63**	**NA**	**54**	**NA**	**18**	**NA**

*Of 196 responders divided by structure

Supplementary Table Q32D. Options ranked 1-3 divided by Specialty

**Option**	**Pathologist**		**Neurosurgeon**		**Neurologist**		**Neuropsychologist**		**Medical Oncologist**		**Radiologist**		**Radiation Oncologist**		**Other**	
	**n**	**%**	**n**	**%**	**n**	**%**	**n**	**%**	**n**	**%**	**n**	**%**	**n**	**%**	**n**	**%**
Promote the collaboration between different specialists in order to foster the culture of multidisciplinarity	11	45.8%	34	36.6%	7	25.9%	0	0%	2	25.0%	0	0.0%	9	37.5%	5	41.7%
Provide a constant platform for the exchange of ideas, useful in clinical practice (e.g., for the management of complex cases) and for the dissemination of information relating to open positions and job opportunities	4	16.7%	27	29.0%	6	22.2%	0	0%	1	12.5%	2	28.6%	9	37.5%	4	33.3%
Promote training initiatives (e.g., point out neuro-oncology meeting, disseminate news from the literature, organize webinars and dedicated sessions in the National Congress)	3	12.5%	20	21.5%	9	33.3%	0	0%	0	0.0%	3	42.9%	5	20.8%	2	16.7%
Promote scientific research initiatives (cooperative studies, collection of case studies, etc.)	11	45.8%	34	36.6%	14	51.9%	1	100%	5	62.5%	4	57.1%	11	45.8%	4	33.3%
Other	3	12.5%	14	15.1%	4	14.8%	0	0%	4	50.0%	2	28.6%	4	16.7%	2	16.7%
**Total***	**24**	**NA**	**93**	**NA**	**27**	**NA**	**1**	**NA**	**8**	**NA**	**7**	**NA**	**24**	**NA**	**12**	**NA**

*Of 196 responders divided by specialty

Supplementary Table Q33. What could be the role of the society AINO in improving neuro-oncology education in Italy?

Option	*n*	%
Promote the inclusion of dedicated neuro-oncology programs in Specialty Schools;	100	51.0%
Promote neuro-oncology training courses within the single-specialty scientific societies	103	52.6%
Promote post-graduate neuro-oncology courses;	120	61.2%
Promote fellowships in the main Italian Neuro-oncology centers	109	55.6%
Other	1	0.5%
**Total***	**196**	**100**

*This was a multiple-choice multiple-answer question. The number of responders was 196.

Supplementary Table Q33A. Divided by age

Option	Age <30		Age 30-39		Age 40-49		Age 50-59		Age >60	
	n	%	*n*	%	*n*	%	*n*	%	*n*	%
Promote the inclusion of dedicated neuro-oncology programs in Specialty Schools;	15	68.2%	53	52.5%	20	41.7%	6	35.3%	6	75.0%
Promote neuro-oncology training courses within the single-specialty scientific societies	11	50.0%	54	53.5%	25	52.1%	9	52.9%	4	50.0%
Promote post-graduate neuro-oncology courses;	10	45.5%	60	59.4%	33	68.8%	11	64.7%	6	75.0%
Promote fellowships in the main Italian Neuro-oncology centers	11	50.0%	57	56.4%	29	60.4%	7	41.2%	5	62.5%
Other	0	0.0%	0	0.0%	1	2.1%	0	0.0%	0	0.0%
**Total***	**22**	**NA**	**101**	**NA**	**48**	**NA**	**17**	**NA**	**8**	**NA**

*Of 196 responders divided by age

Supplementary Table Q33B. Divided by Structure

**Option**	Non-teaching hospital		University Hospital		Scientific Hospital (IRCCS)		Mixed	
	n	**%**	***n***	**%**	***n***	**%**	***n***	**%**
Promote the inclusion of dedicated neuro-oncology programs in Specialty Schools;	29	48.3%	33	52.4%	30	47.6%	7	38.9%
Promote neuro-oncology training courses within the single-specialty scientific societies	33	55.0%	31	49.2%	28	44.4%	10	55.6%
Promote post-graduate neuro-oncology courses;	37	61.7%	33	52.4%	38	60.3%	11	61.1%
Promote fellowships in the main Italian Neuro-oncology centers	35	58.3%	30	47.6%	32	50.8%	11	61.1%
Other	0	0.0%	0	0.0%	1	1.6%	0	0.0%
**Total***	**60**	**NA**	**63**	**NA**	**54**	**NA**	**18**	**NA**

*Of 196 responders divided by structure

Supplementary Table Q33C. Divided by Specialty

**Option**	**Pathologist**		**Neurosurgeon**		**Neurologist**		**Neuropsychologist**		**Medical Oncologist**		**Radiologist**		**Radiation Oncologist**		**Other**	
	**n**	**%**	**n**	**%**	**n**	**%**	**n**	**%**	**n**	**%**	**n**	**%**	**n**	**%**	**n**	**%**
Promote the inclusion of dedicated neuro-oncology programs in Specialty Schools;	13	54.2%	45	48.4%	15	55.6%	1	100.0%	4	50.0%	5	71.4%	11	45.8%	6	50.0%
Promote neuro-oncology training courses within the single-specialty scientific societies	10	41.7%	51	54.8%	13	48.1%	1	100.0%	4	50.0%	5	71.4%	13	54.2%	6	50.0%
Promote post-graduate neuro-oncology courses;	15	62.5%	48	51.6%	15	55.6%	1	100.0%	6	75.0%	7	100.0%	19	79.2%	9	75.0%
Promote fellowships in the main Italian Neuro-oncology centers	16	66.7%	47	50.5%	15	55.6%	1	100.0%	5	62.5%	6	85.7%	12	50.0%	7	58.3%
Other	0	0.0%	0	0.0%	1	3.7%	0	0.0%	0	0.0%	0	0.0%	0	0.0%	0	0.0%
**Total***	**24**	**NA**	**93**	**NA**	**27**	**NA**	**1**	**NA**	**8**	**NA**	**7**	**NA**	**24**	**NA**	**12**	**NA**

*Of 196 responders divided by specialty

Supplementary Table Q34. If you like, please enter a free comment on education in Neuro-Oncology, including aspects not covered in the previous questions, personal experiences, proposals.

**Summary of free comments**
- Role of Continuing Medical Education.<br>- Courses are fundamental but they are expensive. It is necessary to provide scholarships that cover all the expenses.<br>- Increase interdisciplinarity and have more fundings for basic research.<br>- Increase interaction between Institutions.<br>- Parallel paths of theoretical and surgical case-based studies.<br>- Dedicated training modules in the different Specialty Schools involved in management of neuro-oncological patients, or dedicated post-graduate course covering also innovation.<br>- Structured training programs are necessary because Neuro-Oncology is a field in which the management of the patient, which is multidisciplinary, cannot be left to the individual directives of the single specialists. It is necessary to have a multidisciplinary mind at the end of the specialization school.<br>- It is necessary to increase the involvement of smaller Institutions.
